# A post-invasion role for *Chlamydia* type III effector TarP in modulating the dynamics and organization of host cell focal adhesions

**DOI:** 10.1074/jbc.RA120.015219

**Published:** 2020-08-25

**Authors:** António T. Pedrosa, Korinn N. Murphy, Ana T. Nogueira, Amanda J. Brinkworth, Tristan R. Thwaites, Jesse Aaron, Teng-Leong Chew, Rey A. Carabeo

**Affiliations:** 1 Bacteriology Section, Programme in Microbiology, Institute of Medical Sciences, University of Aberdeen, Aberdeen, United Kingdom; 2 Department of Pathology and Microbiology, University of Nebraska Medical Center, Omaha, Nebraska, USA; 3 School of Molecular Biosciences, College of Veterinary Medicine, Washington State University, Pullman, Washington, USA; 4 Advanced Imaging Center, Janelia Research Campus, Howard Hughes Medical Institute, Ashburn, Virginia, USA

**Keywords:** Chlamydia, focal adhesion, pathogenesis, cell adhesion, cell motility, Chlamydia trachomatis, focal adhesions, bacterial pathogenesis, cell adhesion, cell motility

## Abstract

The human pathogen *Chlamydia trachomatis* targets epithelial cells lining the genital mucosa. We observed that infection of various cell types, including fibroblasts and epithelial cells resulted in the formation of unusually stable and mature focal adhesions that resisted disassembly induced by the myosin II inhibitor, blebbistatin. Superresolution microscopy revealed in infected cells the vertical displacement of paxillin and focal adhesion kinase from the signaling layer of focal adhesions, whereas vinculin remained in its normal position within the force transduction layer. The candidate type III effector TarP, which localized to focal adhesions during infection and when expressed ectopically, was sufficient to mimic both the reorganization and blebbistatin-resistant phenotypes. These effects of TarP, including its localization to focal adhesions, required a post-invasion interaction with the host protein vinculin through a specific domain at the C terminus of TarP. This interaction is repurposed from an actin-recruiting and -remodeling complex to one that mediates nanoarchitectural and dynamic changes of focal adhesions. The consequence of *Chlamydia*-stabilized focal adhesions was restricted cell motility and enhanced attachment to the extracellular matrix. Thus, via a novel mechanism, *Chlamydia* inserts TarP within focal adhesions to alter their organization and stability.

Bacterial infection of mucosal epithelial cells triggers the antimicrobial defense strategy of cell exfoliation and apoptosis induction (1). The controlled extrusion of damaged host cells and colonizing pathogens requires the degradation of cell adhesion factors. In epithelial cells, focal adhesions and hemidesmosomes are primarily responsible for attachment to the extracellular matrix, and their assembly and turnover are exquisitely regulated at multiple levels, by kinases, phosphatases, protein-protein interactions, internalization of components, and degradation (2–5). Disruption of one or more of these regulatory processes alters the adhesion dynamics and properties of the cells.

One strategy employed by bacteria to neutralize exfoliation relies on the precise targeting of one or more components of the focal adhesion proteome. The best-characterized example is that of *Shigella*, which neutralizes epithelial extrusion to colonize the epithelium efficiently (6). It does so by delivering the OspE effector by the type III secretion system. This protein reinforces host cell adherence to the basement membrane by interacting with integrin-linked kinase (ILK), a serine/threonine kinase that is part of the focal adhesome (5, 6). A consequence of the OspE-ILK interaction is an increased surface expression of β1-integrin, which in turn promotes focal adhesion (FA) assembly. In addition, the OspE-ILK complex stabilizes the FAs by reducing phosphorylation of focal adhesion kinase (FAK) at a functionally important Tyr-397 residue. Inhibition of this phosphorylation event induces FA disassembly (6). Interestingly, some EPEC and EHEC strains, as well as *Citrobacter rodentium*, possess the effector EspO, which shares strong homology with OspE (7, 8). As such, it is conceivable that these pathogens also reinforce adherence of the infected epithelial cells to secure an infectious foothold. The EspZ effector of EPEC and EHEC has been shown to reduce cell death and detachment *in vitro* (9). EspZ binds the transmembrane glycoprotein CD98 and enhances its effect on β1-integrin signaling and cell survival via activation of FAK (9). It is possible that EspO and EspZ may cooperate to confer enhanced adhesion of the host epithelial cells to the extracellular matrix. Finally, through interaction with human carcino-embryonic antigen-related cell adhesion molecules, bacterial pathogens such as *Neisseria gonorrhoeae*, *Neisseria meningitidis*, *Moraxella catarrhalis*, and *Hemophilus influenzae* can activate β1-integrin signaling and inhibit epithelial cell detachment (1). Despite numerous examples of pathogens manipulating host cell adhesion, the details of these mechanisms remain uncharacterized.

Chlamydiae are obligate intracellular pathogens that are distinguished by their biphasic developmental cycle that alters between the infectious elementary body (EB), and the replicative, but noninfectious reticulate body (RB). At late time points, the noninfectious RBs convert back to EBs to produce infectious particles for the next round of infection. The entire intracellular growth cycle of *Chlamydia* takes ∼48–96 h and occurs within a membrane-bound inclusion, and most of it is spent in the noninfectious RB form. Thus, it is essential that the adhesion of the infected cells to the epithelium is sustained during chlamydial development to enable the differentiation of the noninfectious RBs to the infectious and stable EBs (10). This means that *Chlamydia* must evade a host of antimicrobial defenses, including epithelial extrusion.

Previous works by Kumar and Valdivia (11) and Heymann *et al.* (12) described the loss of motility of *Chlamydia*-infected epithelial cells. Heymann *et al.* (12) attributed this to the chlamydial inhibition of Golgi polarization that occurs at >24 h post-infection, leading to loss of directional migration (12). In this report, we offer an alternate and possibly complementary mechanism of FA stabilization, which could lead to an increase of host cell adhesion to the extracellular matrix (ECM), thus culminating in previously reported loss of motility (11, 12). Using quantitative confocal and live-cell imaging and superresolution microscopy, we describe the various *Chlamydia* infection–dependent changes that occur to FAs consistent with altered cell adhesion, such as increased numbers, enhanced stability, enriched presence of the maturation marker zyxin, resistance to disassembly by the myosin II inhibitor blebbistatin, altered molecular organization, and restricted cell migration. We provide evidence implicating the type III secretion system effector TarP, and its interaction with the focal adhesion protein vinculin, in the majority of these adhesion-related characteristics. We show that vinculin and the region of TarP encompassing binding motifs for focal adhesion kinase and vinculin (LD and VBD, respectively) are required for the localization of the effector to focal adhesions and their resistance to blebbistatin-induced disassembly. We demonstrate that TarP-expressing cells have increased numbers of zyxin-positive focal adhesions. Furthermore, interference-photoactivated localization microscopy (iPALM) reveals that TarP displaces focal adhesion kinase and paxillin from their normal position within the integrin signaling layer. We also show that TarP alone was sufficient to restrict cell motility. Overall, the results indicate that *Chlamydia* has a dedicated mechanism of modulating focal adhesion dynamics through the post-invasion reutilization of TarP and that this process may be linked to the maintenance of *Chlamydia* infection in a high-turnover tissue site.

## Results

### Chlamydia infection enhances FA numbers

**Figure 1.**
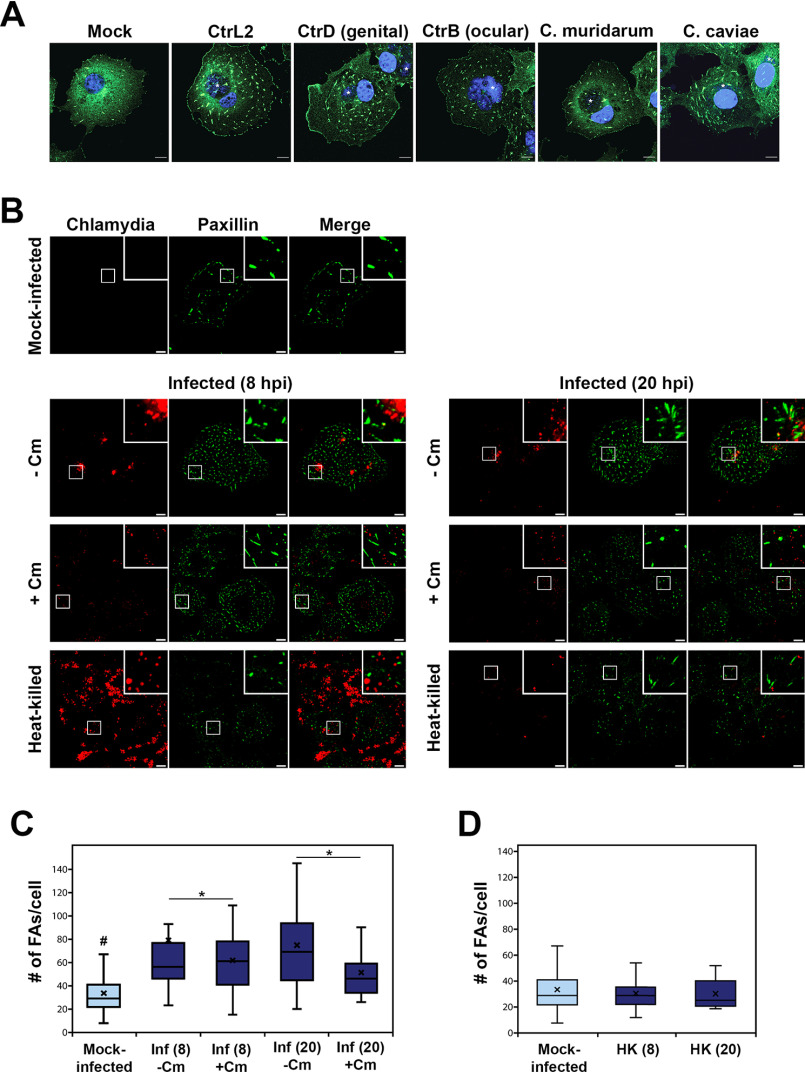
**Infection-dependent increase in focal adhesion numbers requires *de novo* chlamydial protein synthesis.** *A*, COS7 cells infected with the indicated chlamydial strain/species or mock-infected were monitored at 24 hpi, and focal adhesions were visualized by immunostaining for paxillin. The *C. trachomatis* (*Ctr*) serovars used are L2 (lymphogranuloma venereum), D (genital), and B (ocular). *B*, COS7 cells infected with *C. trachomatis* serovar L2 were mock- or chloramphenicol-treated at the start of infection for either 8 or 20 h. Focal adhesions were visualized by immunostaining for paxillin (*green*). *Scale bar*, 10 μm. *C*, focal adhesions were counted using the particle tracker plug-in in NIH ImageJ. Analysis revealed a slight increase in FA numbers in the 8-h Cm treatment group with marginal statistical significance, whereas the 20-h treatment yielded a statistically significant decrease in FA numbers. #, a difference with statistical significance relative to the other groups. *D*, increase in focal adhesion numbers per cell requires viable chlamydiae. Data are represented as *box-and-whisker plots*. *Whiskers* represent the lowest and highest data point still within 1.5 times the interquartile range. The Wilcoxon rank sum test indicated significance. *Black cross*, average for each experimental sample; *, *p* < 0.05.

COS7 cells were infected, and 24 h post-infection (hpi), cells were fixed and prepared for indirect immunofluorescence imaging of paxillin-positive FAs. As shown in Fig. 1*A*, cells infected with *Chlamydia trachomatis* serovar L2, serovar D, serovar B, *Chlamydia caviae* GPIC, and *Chlamydia muridarum* (MoPn) consistently had greater numbers of FAs than mock-infected cells. We further explored the apparent infection-dependent increase in FA numbers using serovar L2 and observed enhanced FA numbers at 8 hpi that increased by 20 hpi. Next, we asked whether the process is pathogen-directed. Specifically, we investigated whether this effect on FAs required *de novo* protein synthesis by *Chlamydia*. COS7 cells were infected with *C. trachomatis* serovar L2 (CtrL2) for the specified duration followed by treatment by the bacterial translation inhibitor, chloramphenicol (Cm). We observed that whereas an 8-h protein synthesis inhibition was not sufficient to prevent the effects on FA number, the 20-h Cm treatment reduced FA numbers of infected cells to the level of mock-infected control (Fig. 1, *B* and *C*). The results indicate that the latter phase of focal adhesion alterations requires either the *de novo* synthesis of new proteins by *Chlamydia* or replenishment of effectors packaged in the metabolically quiescent EBs. These effectors are delivered by the type III secretion system early in infection, prior to differentiation to the vegetative reticulate body form, when they gain the ability for macromolecular synthesis. This is further supported by our observation that heat-killed bacteria could not increase focal adhesion numbers, indicating that viable chlamydiae are essential to this process (Fig. 1*D*).

### The type III effector TarP localizes to FAs in a vinculin-dependent manner and is sufficient to increase FA numbers

**Figure 2.**
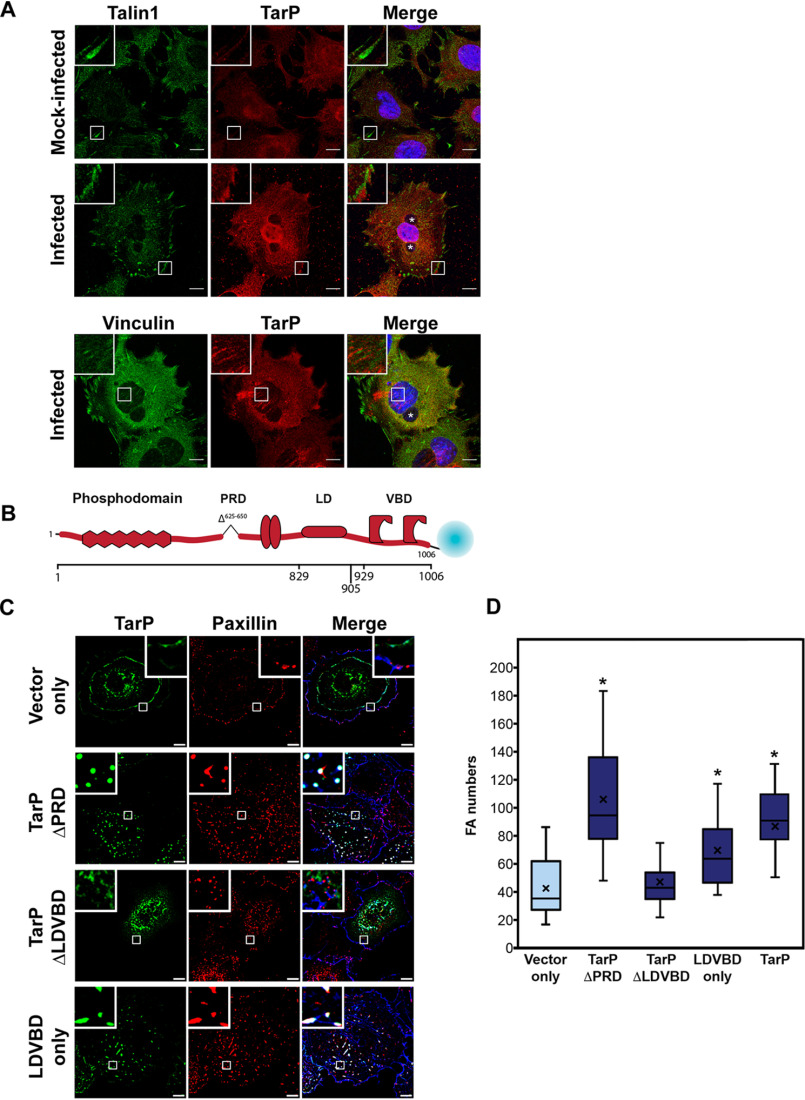
**The type III effector TarP localizes to focal adhesions and is sufficient to increase focal adhesion numbers when ectopically expressed.** *A*, CtrL2-infected MEF cells were immunostained for a rabbit polyclonal antibody to TarP and a mouse mAb to either talin or vinculin. Inclusions were visualized by staining with DAPI and are marked with *asterisks* in the composite image. TarP localized to talin-positive FAs as well as central vinculin-positive FAs in infected, but not in mock-infected controls. *Scale bar*, 10 μm. *B*, representation of *C. trachomatis* effector protein TarP and its known domains fused to mTurquoise2 fluorescent protein. *C*, COS7 cells expressing different deletion derivatives of TarP or vector only were processed for immunofluorescence with anti-paxillin antibody to visualize focal adhesions. Representative images are shown. *Scale bar*, 10 μm. *D*, focal adhesion numbers were counted using the particle-counting plug-in in ImageJ. Data are focal adhesion number per cell and illustrated as a *box-whisker plot*. *Whiskers* represent the lowest and highest data point still within 1.5 times the interquartile range. For statistical analyses, the Wilcoxon rank sum test was used to determine significance when compared with vector-only control (*, *p* < 0.05).

The chlamydial type III effector TarP has been implicated in the invasion process during infection of nonphagocytic cells. Specifically, TarP translocation by the elementary bodies contributes to the actin remodeling that is required for uptake of the pathogen (13–16). Interestingly, TarP was also reported to have a role in increased resistance of infected cells to apoptosis, raising the possibility that this effector has post-invasion function (17). Consistent with this idea is the continuous presence of the protein throughout infection (18) as well as the induction of a second wave of *tarP* transcript (19). In addition, there was sustained presence of TarP translocated to the cytosol as indicated by its reactivity to the anti-phosphotyrosine 4G10 antibody under conditions that prevented further synthesis of this protein (*i.e.* Cm treatment) (Fig. S1). During invasion, TarP localizes to sites of chlamydial adhesion at the plasma membrane (18). If TarP has a role post-invasion, we expect TarP to be found at sites where it exerts its function. Immunostaining with a rabbit polyclonal antibody to CtrL2 TarP of infected mouse embryonic fibroblasts (MEFs) revealed specific staining of FAs, in addition to punctae within inclusions, which are likely to be the bacteria (Fig. 2*A*). Uninfected cells consistently exhibited diffused background immunofluorescence signal, illustrating specificity of the antibody to TarP localized to FAs.

We then sought to determine whether ectopically expressed TarP would yield a similar subcellular localization to FAs. TarP and its deletion derivatives shown in Figs. S2 and S3 were tagged as illustrated in Fig. 2*B* and ectopically expressed in MEFs. TarP has multiple domains that resemble motifs for protein-protein interaction and signaling, including a repeated 50-amino acid domain that is tyrosine-phosphorylated by Src family kinases and the Abl kinase, actin-binding domains, a leucine-aspartate (LD) domain (*e.g.* LD/E*X*LL*XX*L) recognized by the FAK, and vinculin-binding domains (VBDs) (13, 14, 16, 17, 20–24). All have been demonstrated in invasion-related actin recruitment assays to be functional (15, 16, 23). We created various mutant constructs of TarP fused to mTurquoise for heterologous expression in MEFs, as well as a TarP derivative lacking the proline-rich domain (PRD) to minimize nonspecific aggregation of the protein. Transfected cells were counterstained with a mAb to paxillin or FAK to visualize FAs. The extent of TarP localization to focal adhesions was found to correlate with its level of expression, in that higher levels were associated with a greater number of focal adhesions localizing to recombinant TarP. As shown in Fig. 2*C*, colocalization of TarP with the focal adhesion marker paxillin required the LD and VBD motifs. Note that paxillin localization was observed for both full-length TarP and TarP ΔPRD (Fig. S2). In Fig. S3, we showed that proper localization of TarP to FAs required both the LD and VBD motifs. Mutating or deleting one (*e.g.* TarP-LD mut (16) or TarP-ΔVBD) led to the redistribution of TarP, such that it localized along the length of stress fibers or resulted in an outright loss of FA localization (Fig. S3).

**Figure 3.**
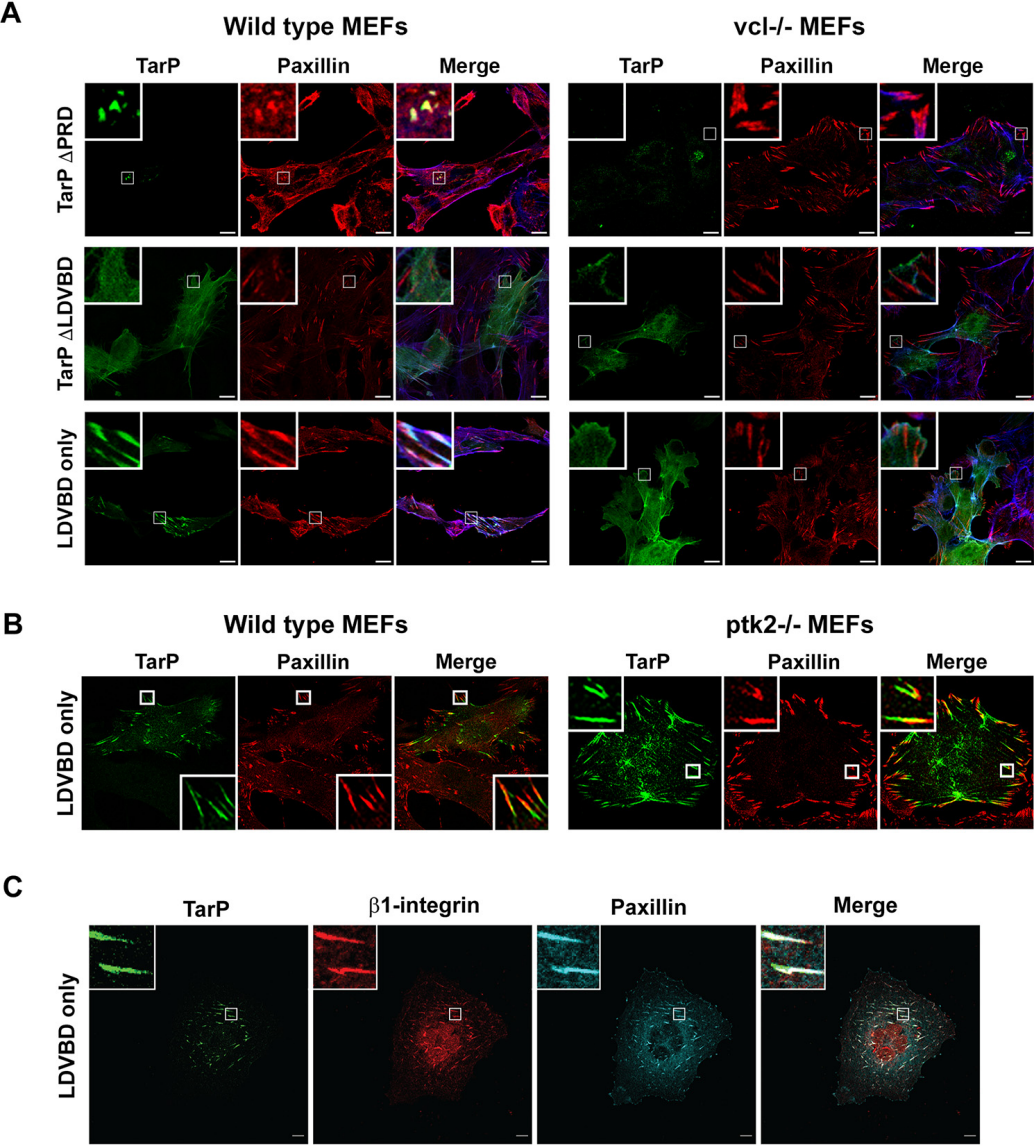
**The focal adhesion localization of TarP requires its LDVBD domain and the host protein vinculin, but not FAK.** *A*, WT or vinculin-knockout MEFs were transfected with different mTurquoise2-tagged TarP constructs (*green*) and imaged by confocal microscopy to evaluate colocalization with paxillin (*red*) at focal adhesions. Phalloidin was used to stain F-actin (*blue*). Cells were transfected for 20 h, at which time the cells were fixed and processed for immunofluorescence staining for paxillin. *B*, in a parallel experiment, WT and FAK-deficient MEFs were transfected to express FLAG-HA-LDVBD and stained for FLAG (*green*). Colocalization with paxillin (*red*) was assessed by confocal microscopy. *C*, ectopically expressed LDVBD localizes to β1-integrin and paxillin-positive focal adhesions. *Scale bar*, 10 μm.

Ectopic expression of TarP or the LDVBD domain led to increased numbers of FAs. Images from the FA localization (Fig. 2*C*) experiments in COS7 cells were reanalyzed by quantifying the number of focal adhesions per cell in cells transfected with the mTurquoise vector alone, TarP ΔPRD, TarP ΔLDVBD, or LDVBD. Paxillin-positive structures in transfected cells were counted in ImageJ (National Institutes of Health), and the data are illustrated as box-whisker plots in Fig. 2*D*. We observed statistically significant increases in focal adhesion numbers in cells expressing TarP ΔPRD and LDVBD. These results suggested that the ability of TarP to localize to FAs, which was mediated by the LDVBD domain of TarP, was linked to its effects on FA numbers.

We previously reported that the VBD and LD domains were recognized by vinculin and FAK, respectively (16, 23). They are distinct nonoverlapping domains that interacted with their respective binding partners independently. Therefore, we evaluated whether the loss of either binding partner (*e.g.* vinculin or FAK) would result in the loss of FA localization. To address the functional relevance of these interactions, albeit in a post-invasion context, the LDVBD construct was expressed in WT, *vcl*^−/−^ (vinculin), or *ptk2*^−/−^ (FAK) MEF knockout mutants. We observed FA localization of mTurquoise-LDVBD in WT MEFs, but not in *vcl*^−/−^ MEFs (Fig. 3*A*). The loss of FAK did not affect the focal adhesion localization of LDVBD (Fig. 3*B*). Together, the data indicated that TarP localization to FAs required the host protein vinculin, likely through its interaction with the VBD domain. FAK was dispensable in this regard. The TarP-positive subcellular structures were verified as FAs based on β1-integrin staining using a mAb specific to the conformationally active form of the receptor (Fig. 3*C*). The LD-dependent FA localization of TarP would suggest a requirement for another host protein that also recognizes this motif, rather than FAK.

### Chlamydia-infected or TarP-expressing cells exhibit vinculin-dependent resistance to blebbistatin

**Figure 4.**
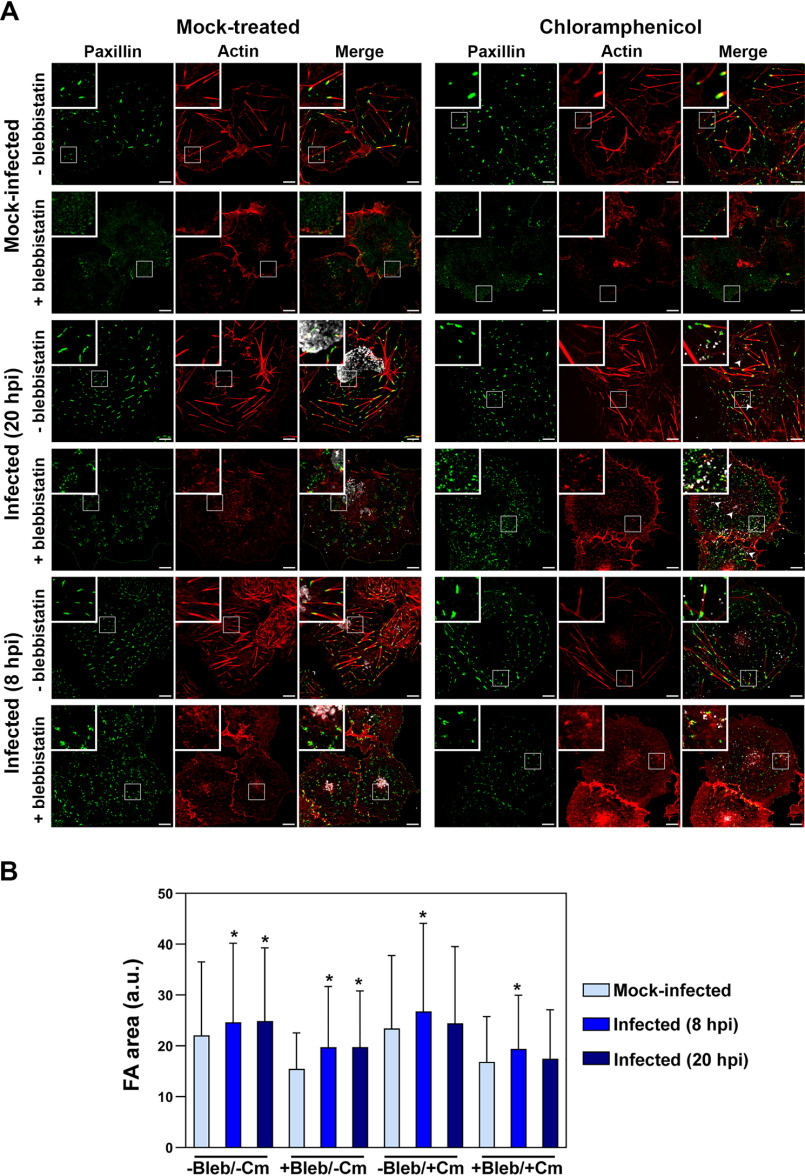
**Focal adhesions of *Chlamydia*-infected cells are resistant to blebbistatin.** *A*, COS7 cells were mock-infected or infected with CtrL2 for 20 or 8 h. Cells were fixed and stained for the focal adhesion marker paxillin (*green*), F-actin (*red*), and human convalescent serum for *C. trachomatis* (*white*). Cells were also mock-treated or pretreated with Cm followed by infection of live EBs to assess the effects of presynthesized EB-associated effectors. Cm treatment was maintained for the duration of the experiment to prevent *de novo* protein synthesis. Blebbistatin (10 μm) was introduced during the last hour of infection. Cells without blebbistatin treatment showed clear F-actin stress fibers and paxillin-labeled focal adhesions. Whereas both structures were lost in mock-infected cells, infected cells retained the focal adhesions. *Scale bar*, 10 μm. *B*, images were run through the Focal Adhesion Analysis Server to obtain area values. For each treatment, >850 FAs were analyzed. The *bar graph* represents means with S.D. Statistical significance was assessed using a Kruskal–Wallis test followed by a post hoc Dunn's test. *, significance (*p* < 0.01) relative to the mock-infected sample for each treatment.

A marked difference during infection was the increased number of FAs at the interior of the cell as evidenced in Fig. 1 Focal adhesion maturation is associated with movement away from the cell periphery and toward the center (25). FAs at the interior of the cell either mature to become stable fibrillar adhesions to promote cell attachment or disassemble during migration (25–27). We speculated that the increased numbers of interior FAs arose from infection-dependent stabilization. To assess stability, we took advantage of the enhanced turnover of FAs in the presence of the myosin II–specific inhibitor, blebbistatin (28). FA stability depends on tension within and between focal adhesions (29–31). This tension is largely provided by the contractile action of the molecular motor myosin II on stress fibers (SFs), and its inhibition by blebbistatin consistently leads to FA disassembly and altered motility (26, 32, 33). We evaluated the relative resistance of FAs to a 60-min treatment with 10 μm blebbistatin in the context of infection with CtrL2 at 20 hpi. As shown in Fig. 4*A* of representative samples, SFs in the mock-infected cell disassembled, with the simultaneous disappearance of FAs marked with paxillin. In contrast, FAs in the CtrL2-infected cells remained, whereas infection did not prevent blebbistatin-induced disassembly of stress fibers. The latter observation confirmed blebbistatin's inhibitory activity. Taken together, the results point to a dedicated mechanism in *C. trachomatis* and perhaps other chlamydial species to stabilize focal adhesions, as indicated by their resistance to blebbistatin. In addition, our observations indicated that *Chlamydia* could uncouple focal adhesion stabilization from stress fibers. Interestingly, chloramphenicol treatment had no effect on *Chlamydia*-conferred blebbistatin resistance, except for the 20-h treatment group (Fig. 4*B*), indicating that prepackaged chlamydial protein, of which TarP is one (Fig. S1), is sufficient to confer partial stability to focal adhesions. However, we did observe a reduction in the average size of the remaining focal adhesions post-blebbistatin treatment for all groups (*e.g.* mock infection and 8 and 20 h post-infection), but infection blunted the effect of the inhibitor when compared with mock-infected, blebbistatin-treated samples. Chloramphenicol treatment attenuated blebbistatin resistance of focal adhesions of the infected cells for the 20-h group (Fig. 4*B*). Overall, the data point to the existence of a presynthesized EB-associated factor that confers to focal adhesions a level of resistance to blebbistatin-induced disassembly, and this factor needed to be replenished during infection.

**Figure 5.**
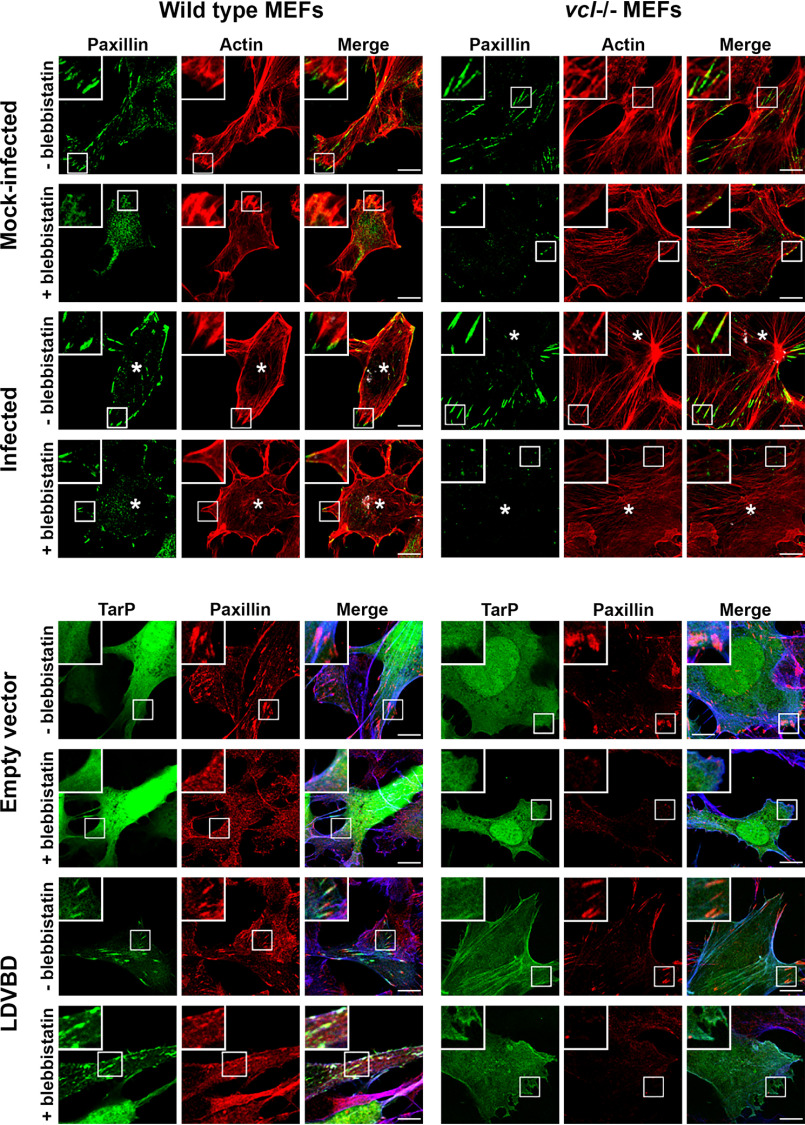
**The LDVBD domain of TarP and the host protein vinculin are required for focal adhesion resistance to blebbistatin treatment.** *Top*, WT or vinculin-knockout MEFs were infected with *C. trachomatis* serovar L2. Cells at 20 hpi were mock-treated or treated for 60 min with 10 μm blebbistatin. The cells were then processed for immunofluorescence staining for paxillin (*green*) and actin (*red*). Retention or loss of focal adhesions was monitored. Focal adhesions were only resistant to blebbistatin-induced disassembly if the cell was infected and expressing vinculin. *Bottom*, in a parallel experiment, WT or vinculin-knockout MEFs were transfected for 20 h with the empty vector or LDVBD-mTurquoise2 fusion protein. During the last hour, cells were either mock- or blebbistatin-treated. Cells were processed to visualize paxillin (*red*), LDVBD (*green*), and actin (*blue*; shown in *composite images*). LDVBD was sufficient to confer resistance to blebbistatin-induced disassembly to focal adhesions. Resistance also required vinculin. *Scale bar*, 10 μm.

We also evaluated the effect of ectopically expressed LDVBD on the stability of focal adhesions, specifically if its vinculin-dependent localization to FAs (Fig. 3*A*) was required for resistance to disassembly by blebbistatin. First, we wanted to establish the role of vinculin in blebbistatin resistance of FAs in the context of infection. As illustrated in Fig. 5 (*top*), *Chlamydia*-infected WT MEFs retained paxillin-marked FAs after 60 min of treatment with 10 μm blebbistatin, whereas mock-infected cells lost them. In contrast, infection of the *vcl*^−/−^ MEFs failed to inhibit blebbistatin-induced disassembly of focal adhesions, highlighting the crucial role of the host protein vinculin in FA stability. We then investigated whether the LDVBD domain was sufficient to confer a similar resistance to blebbistatin-induced disassembly and whether it did so in a vinculin-dependent manner. LDVBD transfection of WT MEFs led to the retention of FAs after the 60-min treatment with blebbistatin, whereas the *vcl*^−/−^ MEFs lost these structures despite LDVBD expression (Fig. 5, *bottom*). Therefore, we concluded that vinculin plays an important role in TarP-dependent stabilization of FAs. Whether the apparent stabilizing role of vinculin is due to the FA localization of TarP or an alteration of its activity as a result of its interaction with TarP remains to be addressed.

### Blebbistatin-resistant focal adhesions are positive for the maturation marker zyxin

**Figure 6.**
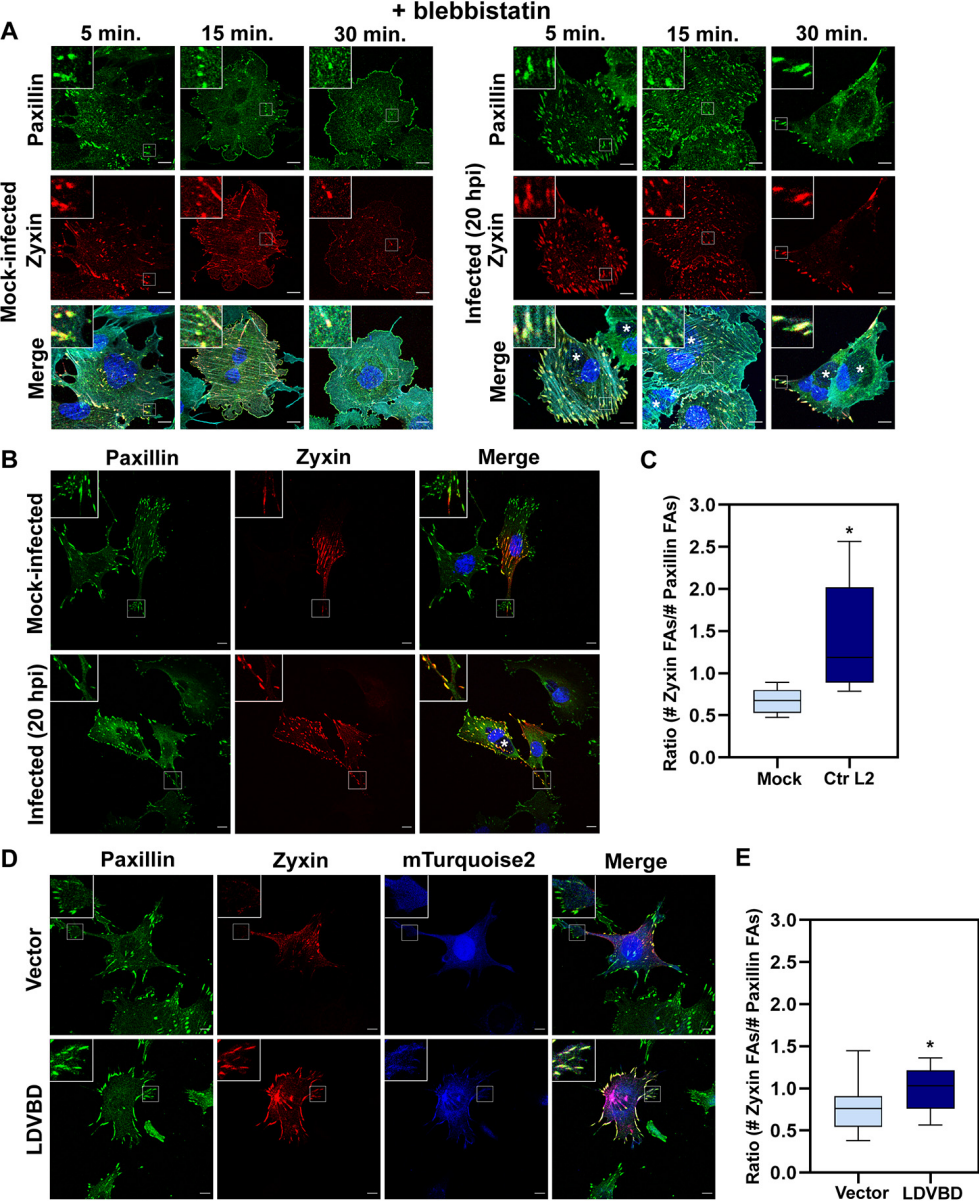
**An increased number of FAs contain the maturation marker zyxin in *Chlamydia*-infected and LDVBD-expressing cells.** *A*, MEF cells were transfected with RFP-zyxin via electroporation and then mock-infected or infected with CtrL2 for 20 h. Cells were treated with 10 μm blebbistatin and fixed at the indicated time points and then stained for the focal adhesion marker paxillin (*green*) as well as actin (*cyan*; shown in *composite images*). *Chlamydia*-containing inclusions were visualized by staining with DAPI and are marked with *asterisks* in *composite images*. *B*, CtrL2-infected MEF cells expressing RFP-zyxin were processed for immunofluorescence with anti-paxillin antibody to visualize focal adhesions. Inclusions were visualized by staining with DAPI. Representative images are shown. *Scale bar*, 10 μm. *C*, the paxillin and zyxin channels were submitted to the Focal Adhesion Analysis Server to obtain a mask of each channel. The focal adhesion number was then counted using the particle-counting plug-in from ImageJ. Data are the number of zyxin adhesions present divided by the number of paxillin adhesions present per individual cell and illustrated as a *box-whisker plot*. *Whiskers* represent the lowest and highest data point still within 1.5 times the interquartile range. For statistical analyses, ANOVA was used to determine significance when compared with a mock control (*, *p* < 0.001) (*n* = 10 cells). *D*, MEF cells expressing RFP-zyxin as well as vector only or LDVBD-mTurquoise2 were processed for immunofluorescence with anti-paxillin antibody. Representative images are shown. *E*, quantification performed as described in *C*. For statistical analyses, ANOVA was used to determine significance when compared with vector-only control (*, *p* < 0.05) (*n* = 10 cells).

In addition to movement away from the cell periphery, focal adhesion maturation is characterized by the presence of specific FA components within the adhesome. Because FAs form via the sequential incorporation of multiple protein components, their protein composition varies during different stages of development (34). As a result, newly forming FA complexes can be distinguished from mature FAs based on the presence or absence of specific protein markers. New FAs contain talin, paxillin, and low levels of vinculin and FAK (35). However, zyxin incorporates into FAs later in their maturation process and is typically associated with a relative increase in stability. In response to tension, zyxin will redistribute along stress fibers, ultimately reinforcing focal adhesion stability (36, 37). This makes zyxin an ideal marker for tension-dependent maturation of an adhesion. We decided to further investigate the blebbistatin resistance phenotype by evaluating the maturation state of the adhesions that remained. To accomplish this, we treated mock- or CtrL2-infected cells with 10 μm blebbistatin and fixed samples at 5, 15, or 30 min to examine whether the remaining adhesions were zyxin-positive. If mature adhesions are more stable, and thus more resistant to blebbistatin, we would expect to see zyxin-positive adhesions predominate and be the last to disassemble during the time course. As shown in Fig. 6*A*, focal adhesions of mock-infected cells start to rapidly disassemble as early as the 15-min mark, whereas focal adhesions of CtrL2-infected cells remain stable throughout the series. Notably, representative figures of paxillin- and zyxin-marked focal adhesions show that the remaining adhesions for both mock and CtrL2-infected cells contain zyxin. This suggests that the mature, zyxin-positive adhesions are most resistant to blebbistatin-induced disassembly. Strikingly, we observed that CtrL2-infected cells contain adhesions that almost exclusively contain both paxillin and the maturation marker zyxin. Comparatively, in mock-infected cells there appeared to be more adhesions containing only paxillin than adhesions positive for both markers.

To follow up on this phenotype, we evaluated the maturation state of adhesions within CtrL2-infected cells outside of the context of blebbistatin resistance, using zyxin as our marker. As shown in Fig. 6*B*, CtrL2-infected WT MEF cells exhibit increased numbers of zyxin-containing adhesions compared with a mock-infected control. During infection, we observed that even small, likely nascent adhesions present at the leading edge of the cell contained zyxin. These data were quantified in Fig. 6*C* by calculating the ratio of the number of zyxin-positive adhesions over the number of paxillin-positive adhesions present in an individual cell. We found that LDVBD-expressing cells also exhibited an increase in the number of zyxin-positive focal adhesions present compared with vector alone (Fig. 6, *D* and *E*). Compared with their respective controls, CtrL2-infected cells showed a larger increase than LDVBD-expressing cells. It is possible additional chlamydial factors are required to fully alter the maturation state of host cell adhesions or that changes in intracellular tension during infection may play a role, as zyxin has been shown to be a mechanosensitive protein. Overall, these findings suggest to us that *Chlamydia* may take advantage of the natural enhanced stability of mature focal adhesions to facilitate increased host cell adherence. By increasing the proportion of mature adhesions within a cell, the pathogen can exploit the naturally occurring process of adhesion maturation to promote enhanced adherence of the host cell.

### Infection disrupts the stratified organization of focal adhesions, a phenotype mimicked by the ectopic expression of TarP

**Figure 7.**
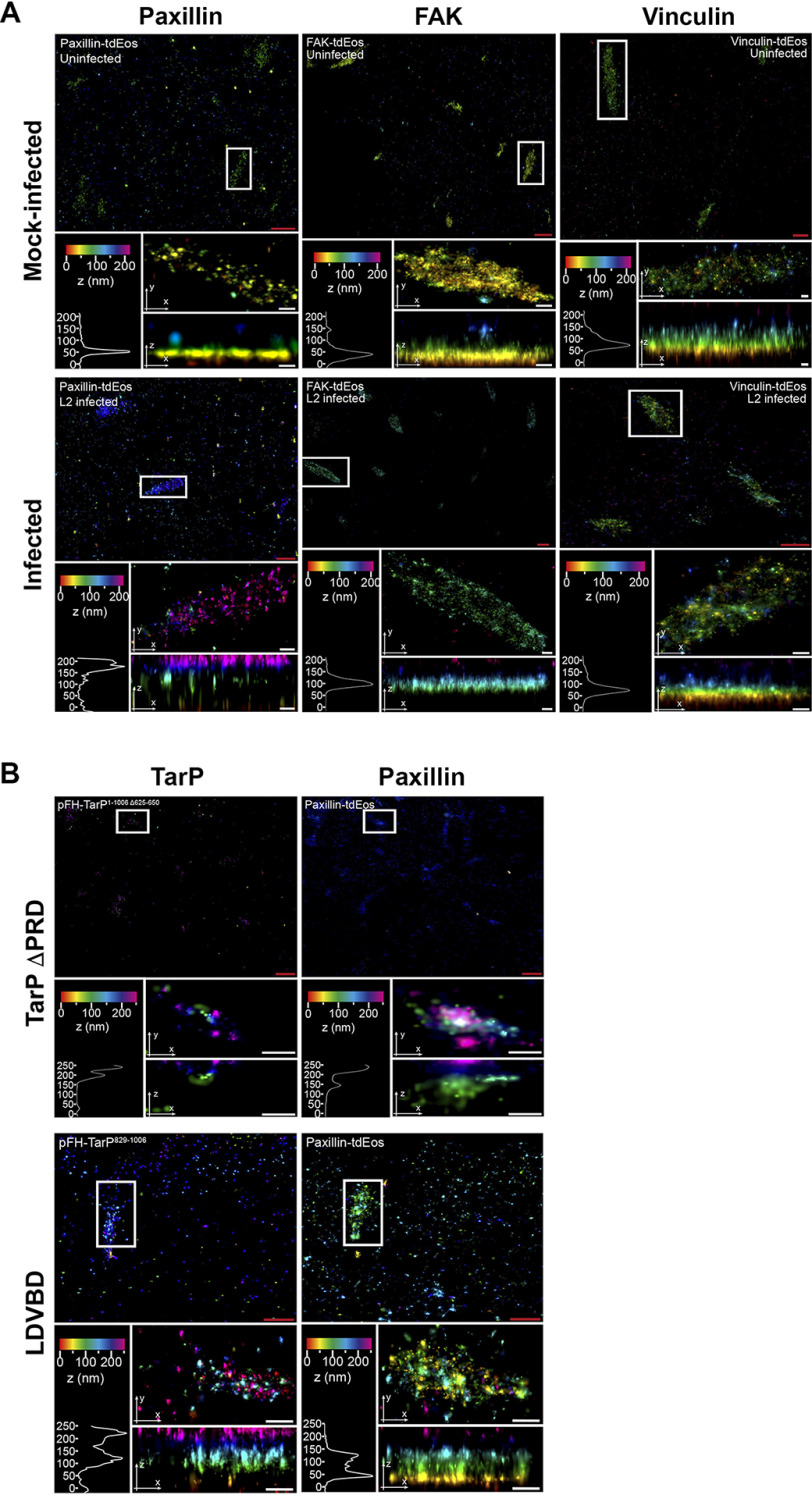
**TarP-targeted focal adhesions display altered nanoscale architecture.** *A*, COS7 cells were pretransfected with paxillin-tdEos, FAK-TdEos, or vinculin-TdEos on gold fiducial coverslips and were mock-infected or *C. trachomatis*–infected for 20 h. The cells were fixed and processed for iPALM imaging. Representative images are shown from *n* = 3. For each sample, multiple *panels* are provided. The *top panel* shows the *top view* of the area around the focal adhesion of interest (*white border*). The *middle panel* displays a *top view* of the focal adhesion indicated by the *white border*. The *bottom panel* shows the *side view* and corresponding *z* histograms. Note the significant shifts in paxillin and FAK localization, but not vinculin. *B*, COS7 cells were co-transfected with paxillin-tdEos and either TarP ΔPRD or LDVBD only by electroporation. The cells were seeded on gold fiducial coverslips and processed for iPALM at 24 h post-transfection at *n* = 2. Description of each *panel* is as above in *A*. Note the significant shift in the location of paxillin within the TarP-positive focal adhesions. The various *colors* indicate the distance (*z*-coordinates) from the gold fiducial marker (*e.g.* z = 0 nm; *red*). *Red scale bar*, 1 μm. *White scale bar*, 200 nm.

Focal adhesions are organized into distinct strata termed the integrin layer, the signaling layer, which harbors paxillin and FAK among others, and the force transduction layer that contains vinculin, talin, and other mechanosensitive proteins. At the highest layer, actin and actin-associated proteins, such as α-actinin and myosin II are found (38, 39). Given the profound effect of infection and TarP ectopic expression on FA stability, we investigated using iPALM their effects on FA organization as described under “Materials and methods.” The localization of paxillin, FAK, and vinculin, all fused to mTurquoise2, was monitored in mock-infected, CtrL2-infected, TarP ΔPRD, or LDVBD-transfected cells (Fig. 7). Image analysis revealed dramatic reorganization of FAs with regard to paxillin and FAK. In representative control samples, paxillin, FAK, and vinculin were found 50.5, 40.4, and 71 nm from the bottom of the cell, respectively, consistent with previous findings (38). However, paxillin and FAK shifted upward to 176.9 and 96 nm, respectively, in representative infected cells, whereas no change in location was observed for vinculin (Fig. 7*A*), which points to the specific disruption of FA organization by *Chlamydia*. The state of infection of cells analyzed is shown in Fig. S4.

Expression of TarP ΔPRD also caused a shift (240.2 nm) in paxillin localization (Fig. 7*B*). LDVBD expression caused a noticeable shift (47 nm, with a second peak at 130 nm), but to a lesser degree than TarP ΔPRD, indicating that additional elements of TarP absent in the LDVBD are necessary.

### Cell motility is restricted in Chlamydia-infected or TarP-expressing cells

**Figure 8.**
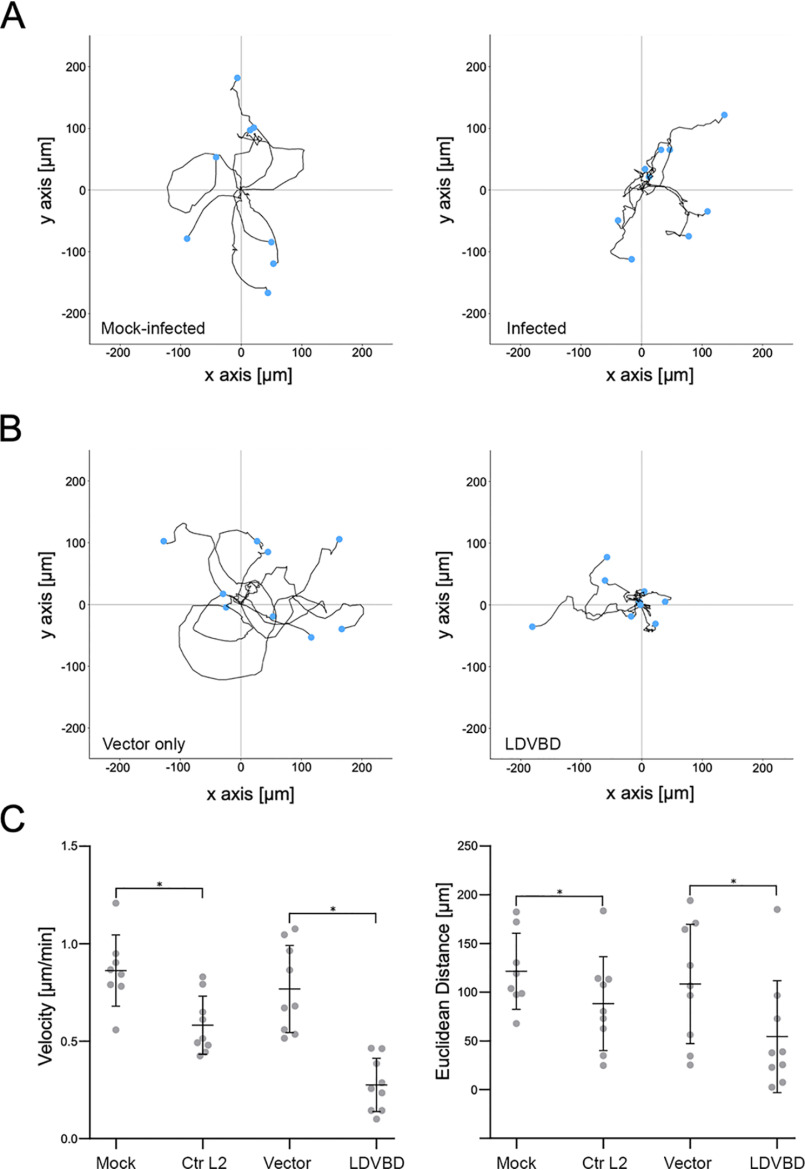
**The LDVBD domain of TarP is sufficient to inhibit cell migration.** MEFs that were mock-infected, *Chlamydia*-infected, vector-only–transfected, or LDVBD-transfected were seeded within ibidi µ-slide live-cell imaging chambers. Time-lapse imaging was performed every 10 min for 10 h to evaluate cell motility. *A*, for analysis of the infection experiments, a 5-h imaging window common to both mock- and *Chlamydia*-infected samples was chosen that maximized the number of cells that remained within the field of view. Cells were tracked using the manual tracking function in ImageJ, and the cell trajectory was traced and plotted with the starting points assigned to the origin. *B*, analysis of the transfection experiment was in a common 10-h imaging window. Data were acquired and plotted as in *A*. *C*, velocity and Euclidean distance traveled were calculated for each cell from each experimental group. Values were plotted as dot plots with mean ± S.D. indicated by the *bars*. Statistical significance was calculated using ANOVA. *, *p* < 0.01.

It was previously reported that *Chlamydia*-infected cells were restricted in their motility, and this was attributed to the inability of infected cells to establish front-rear polarity due to Golgi fragmentation induced by the pathogen during late stages of infection (12). We decided to reinvestigate the loss of motility of infected cells by focusing on focal adhesion dynamics, which was shown in Fig. 1*B* to occur as early as 8 hpi. The decision of the cell to migrate or adhere involves the regulation of focal adhesion stability in response to external cues, such as chemoattractants and ECM stiffness. First, we verified that *Chlamydia*-infected mouse embryo fibroblasts were severely limited in their ability to migrate, relative to mock-infected control cells (Movies S1 and S2). The manual tracking plug-in of ImageJ was utilized to obtain cell trajectory tracks for the motility assay. Cells were tracked using the position of the nucleus over time. The coordinate data were input into ibidi's chemotaxis and migration tool to obtain velocity and distance measurements. Velocity measurements revealed a 1.5-fold decrease in the mean rate of migration of infected cells (Fig. 8, *A* and *C*).

Mouse embryonic fibroblasts were transfected to ectopically express mTurquoise2 vector only or the LDVBD domain. Cell migration was monitored in the DIC channel with fluorescence images taken at the end of the motility assay (Movies S3 and S4 and Fig. S5). To quantify motility, the cells were tracked as described above, with accompanying velocity calculations. As shown in Fig. 8 (*A* and *B*), infected and LDVBD-transfected cells were significantly restricted in their motility relative to mock-infected or vector-transfected controls. Both distance and velocity of LDVBD-transfected cells were further restricted to those of infected cells (Fig. 8*C*), indicating that inhibition of cell migration by *C. trachomatis* could be accounted for fully by TarP overexpression. The enhanced inhibition of migration distance and velocity in transfected cells may have been due to increased levels of ectopically expressed LDVBD when compared with levels present during infection.

### Infection by Chlamydia trachomatis but not ectopic expression of TarP confers resistance to detachment by mild trypsinization

Exfoliation of epithelial cells from the infected epithelium has been reported in rodent models of ocular and genital infection, and both reports speculated on the involvement of neutrophil-derived proteases in the process (40, 41). We evaluated the resistance of infected epithelial cells to detachment by 0.025% trypsin and monitored for cell rounding by time-lapse imaging at 1-min intervals for 30 min (Movies S5 and S6). Uninfected HeLa cells started detaching by 7 min post-trypsinization, whereas *C. trachomatis* L2–infected cells remained attached through the duration of imaging (30 min after addition of trypsin). To evaluate whether TarP ectopic expression would be sufficient to resist detachment, the cells were transfected for 24 h to overexpress (LDVBD-mTurquoise2). Monolayers were imaged under fluorescence microscopy at 0, 15, and 30 min after trypsinization. If TarP LDVBD overexpression was sufficient to induce detachment resistance, we would expect an enrichment in remaining adherent cells of those expressing LDVBD-mTurquoise2 compared with cells expressing mTurquoise2 only. Percentage values for both LDVBD-mTurquoise2 and vector-only samples were 11.9% *versus* 11.0% prior to trypsinization. We obtained the following for LDVBD *versus* vector-only: 10.2% *versus* 10.3% (15 min) and 8.7% *versus* 11.0% (30 min). No statistically significant differences were found between LDVBD and vector-only for either trypsinization time point. These results indicated that, whereas TarP is able to reduce cell motility, it was not sufficient to confer resistance to detachment by mild trypsinization. We interpret this to mean that additional changes to focal adhesions, possibly mediated by additional chlamydial factors, are required.

## Discussion

All chlamydial species, to varying extents, exhibit tropism to epithelial cells and thus likely evolved to counteract cell extrusion associated with the normal cycle of turnover of the epithelium or as an antimicrobial mechanism to limit dissemination and eliminate infection. The latter may also involve polymorphonuclear cells, which secrete proteases to degrade adhesion structures of epithelial cells. Thus, *Chlamydia*, with its biphasic developmental cycle that involves a temporary loss of infectivity, is subjected to a very strong selective pressure to acquire mechanisms to inhibit epithelial cell extrusion. A large portion of the developmental cycle is spent in the noninfectious form, and thus, it is crucial to the survival of the pathogen to inhibit extrusion of host epithelial cells before *Chlamydia* can convert to the infectious form. Here, we demonstrated that adhesion of infected cells is enhanced via the action of TarP, an effector protein conserved in the genus *Chlamydia*, and the host cell protein vinculin.

Our data collectively point to FAs as targets for modulation by *Chlamydia*. The type III effector TarP plays a role in this modulation. The mechanism involves the vinculin-dependent localization of TarP to FAs. Whereas the interaction between FAK and TarP did not seem to be essential to the focal adhesion localization of the chlamydial effector, it remains possible that the LD domain contributes to focal adhesion stability by facilitating the localization of TarP at FAs, where it could interact with other proteins and/or interfere with the interactome of LD-harboring focal adhesion proteins like paxillin through competition. The presence of LDVBD at FAs leads to maturation and stability, the former supported by findings of increased zyxin-positive FAs in the presence of LDVBD. Within the adhesion, there were instances of the localization of TarP and two markers (*e.g.* vinculin and talin) being offset in a proximal-distal orientation. Similar observations have been reported for other focal adhesion markers, specifically during sensing of extracellular matrix stiffness, where paxillin relocalized to the proximal end of the focal adhesion relative to the resident vinculin. In the case of TarP, it would be intriguing if its position within focal adhesions is regulated by tension (42).

We also made the novel observation of FA reorganization in *Chlamydia*-infected or TarP-expressing cells, with paxillin and FAK displaced from the signaling layer. A pressing question is whether the reorganization is the cause or the effect of enhanced stability of TarP-targeted FAs. Considering the reported relative stability of FAs in FAK-depleted cells (43), it is possible that the displacement of FAK by TarP could be analogous to a loss of function. A more comprehensive investigation of FA disorganization by TarP is required to define the exact mechanism of FA stabilization by this chlamydial effector. FAK displacement might negatively affect interactions with signaling proteins, like Src kinase, thus disrupting the progression of tyrosine phosphorylation along the FAK protein, a process crucial to disassembly of FAs. Paxillin was similarly displaced in both infected and TarP-expressing cells, which likely disrupts protein-protein interactions and signaling related to paxillin within FAs. To our knowledge, FA reorganization to the extent that we observed in infected cells has not been reported, pointing to a novel mechanism of regulating FA dynamics.

Whereas TarP, specifically the LDVBD domain, was sufficient to drive FA stability to inhibit cell motility, it was unable to mimic the resistance of infected cells to detachment by mild trypsinization, which was meant to replicate polymorphonuclear cell–mediated extrusion of infected epithelial cells. This would be consistent with the involvement of additional chlamydial factors that may mediate various aspects of focal adhesion characteristics, in addition to numbers. Additional factors may facilitate progression of maturation, possibly to fibrillar adhesions. Another might be the infection-dependent production of extracellular matrix components by the host cell, including collagen. Increased deposition of collagen underneath infected cells would likely influence FA stability by engaging integrins and inducing “outside-in” FA-stabilizing signals. Thus, *Chlamydia* might have multiple cooperating mechanisms ensuring the strong adhesion of its host cell, highlighting the importance of counteracting epithelial cell extrusion.

We speculate that FA stabilization might be one mechanism by which *Chlamydia* neutralize extrusion of epithelial cells. It is known that this process could limit infection dissemination during infection for a number of epitheliotropic pathogens. Shedding of epithelial cells from uropathogenic *Escherichia coli* (UPEC)-infected bladder is thought to reduce bacterial burden, facilitating resolution of infection (44). The intestinal pathogen *Shigella* possesses an effector, OspE that modulates epithelial cell attachment to facilitate the pathogen's cell-to-cell spread. Indeed, loss of OspE resulted in a significant decrease in virulence (1, 6). Epithelial cells of the genital tract or the ocular mucosa also experience higher rates of turnover and are constantly replenished (45–47). The process of epithelial extrusion is complex. In addition to promoting detachment of the cells from the ECM, proper extrusion requires that interactions with neighboring cells via disassembly of intercellular junctions must be regulated to maintain epithelial barrier integrity (4, 48). Stabilizing FAs could also promote increased resistance to apoptosis. Cell detachment is associated with anoikis, a programmed cell death associated with loss of adherence (49, 50). Focal adhesions provide prosurvival/anti-apoptotic signals (51), and stabilization of these structures would clearly benefit obligate intracellular pathogens like *Chlamydia*.

An interesting question is the means by which epithelial cell extrusion is triggered. Is it part of the normal cell turnover during tissue remodeling/renewal, or is it linked to pathogen recognition? The shedding of bladder epithelial cells during UPEC infection requires the expression of bacterial type 1 pili, which is a potent pathogen-associated molecular pattern that is recognized by the Toll-like receptor 4 (TLR4) (42), raising the intriguing possibility of a direct link between regulation of cell adhesion dynamics and pathogen recognition. Various chlamydial species are recognized by Toll-like receptors expressed on epithelial cells (*e.g.* TLR2, TLR3, TLR4, and TLR9) (52–58), which lends credence to cell extrusion being triggered by pathogen recognition.

To date, the fundamental question of how *Chlamydia* is able to colonize a tissue site with relatively high cell turnover has received little attention. Here we provided evidence for the existence of a mechanism that *C. trachomatis* potentially relies on during *in vivo* infection of the genital mucosa. We identified an infection-dependent change to host cells (FA stability, FA reorganization, and restricted cell motility), a type III effector (TarP) responsible, the relevant target (FAs) of the effector, and an initial mechanism (vinculin dependence). Taken together, our observation that cells infected with various chlamydial species all exhibited increased FA numbers and the conservation of TarP, specifically the LDVBD domain, across species indicate that this mechanism is a pan-chlamydial strategy for counteracting cell turnover. Furthermore, this process is mechanistically distinct from those of other epitheliotropic pathogens, thus expanding the repertoire of strategies designed to neutralize epithelial cell extrusion.

## Materials and methods

### Cell culture

COS7 (ATCC CRL-1651), NIH3T3 (kindly supplied by Hector Aguilar-Carreño ATCC CRL-1658), and HeLa 229 (ATCC CCL-2.1) were used in this paper. MEFs *vcl*^−/−^ and matched MEFs *vcl*^+/+^ (59) were kindly provided by Dr. Wolfgang Ziegler (Hannover Medical School). Cells were cultured using Dulbecco's modified Eagle's medium (DMEM) (Thermo Fisher Scientific, 11960-085). Media were supplemented with 10% fetal bovine serum (Sigma, F0804-500ML), 2 mm l-glutamine, and 10 μg/ml gentamicin. *C. trachomatis* serovar L2 (L2/434/Bu) was propagated in HeLa 229. EBs were harvested by discontinuous density gradient centrifugation in Gastrografin (Bracco Diagnostics), as described previously (16).

### Chlamydia infections

Cells were infected with *C. trachomatis* serovar L2 (L2/434/Bu, CtrL2) at a multiplicity of infection (MOI) of 5 for 20 h and at an MOI of 25 for 8 h in ice-cold serum-free DMEM. Cells were centrifuged at 1000 rpm for 15 min at 4 °C to synchronize the infection. After centrifugation, the inoculum was replaced with warm DMEM supplemented with 10% fetal bovine serum, 2 mm l-glutamine, and 10 μg/ml gentamicin. In parallel, a mock-infected control was made following the same protocol but without *Chlamydia* infectious particles. Infection by other strains/serovars was as follows. COS7 cells were grown on glass coverslips. Cells were infected with *C. trachomatis* serovar L2, serovar D, serovar B, *C. muridarum* (MoPn), or *C. caviae* (GPIC) at an MOI of 5 for 24 h. Cells were centrifuged at 500 × *g* for 15 min at 4 °C to synchronize the infection. A mock-infected control was made following the same protocol but without *Chlamydia* infectious particles. Prior to infection with serovar D, cells were pretreated with 1× DEAE-dextran for 15 min at room temperature. Pretreatment was followed by two washes with 1× Hanks' balanced salt solution and replacement with DMEM to continue the infection.

### Immunostaining

Cells were grown on fibronectin-coated coverslips (Neuvitro, GG-12-fibronectin) for the duration of the experiment. At a predetermined time, cells were rinsed with Hanks' balanced salt solution (Thermo Fisher Scientific, 14025-100) and fixed using 4% paraformaldehyde (PFA) in PBS, pH 7.4 (Gibco, 14190-094) for 20 min at room temperature. The fixed cells were then permeabilized using PBS with 0.2% Triton X-100. Subsequently, permeabilized cells were incubated with 1% BSA (Sigma, A9418) in PBS for 30 min at room temperature to block nonspecific antigen binding. Cells were then incubated with the primary antibodies overnight at 4 °C with rocking. The primary antibodies used in this study were rabbit polyclonal antibody against FAK phosphorylated at tyrosine 397 (pFAK-Y397) (Abcam, ab4803), rabbit mAb paxillin (Abcam, ab32084), mouse mAb vinculin (Abcam, ab18058), rat monoclonal 9EG7 against the active form of β1-integrin (BD Biosciences, 553715), mouse monoclonal FLAG tag antibody (Cell Signaling, 8146S), mouse mAb *Chlamydia* LPS (Abcam, ab62708), and convalescent human sera. Afterward, cells were incubated with appropriate fluorescently conjugated secondary antibodies and, when specified, with DAPI (Roche Applied Science, 10236276001) and Alexa Fluor 488 phalloidin stains for 1 h at room temperature with rocking. In this study, the following secondary antibodies were used: goat anti-rabbit Alexa Fluor 488 (Thermo Fisher Scientific, A11008), goat anti-rabbit Alexa Fluor 633 (Thermo Fisher Scientific, A21071), goat anti-mouse Alexa Fluor 594 (Thermo Fisher Scientific, A11005), goat anti-human Alexa Fluor 647 (Thermo Fisher Scientific, A-21445). Following staining, the coverslips were mounted with Mowiol and visualized in a Zeiss LSM 710 confocal microscope in the Microscopy and Histology Core Facility at the University of Aberdeen or in the Leica SP8 confocal microscope at the Washington State University Integrative Physiology and Neuroscience Imaging Core. Fiji software (60, 61) was used to generate the final images. Image-processing parameters were kept constant for all related samples.

To localize endogenous TarP to focal adhesions, MEFs grown on glass coverslips were infected with *C. trachomatis* L2 at an MOI of 10 for 20 h. Cells were centrifuged at 500 × *g* for 15 min at 4 °C to synchronize the infection. A mock-infected control was made following the same protocol but without *Chlamydia* infectious particles. Cells were fixed using ice-cold 100% methanol for 1 min. Cells were then blocked with 5% BSA for 1 h at room temperature. Focal adhesions and TarP were visualized respectively using a primary monoclonal Talin1 antibody (Novus Biologics, NBP2-50320) and rabbit polyclonal TarP antibody generated against the epitope (amino acids 661–710) (Li International, Denver, CO, USA). Samples were incubated overnight at 4 °C. Immunostaining with secondary antibodies was as described above.

### Time-lapse microscopy

For live-cell imaging of FAs, fibroblasts were seeded on ibidi μ-slide 8-well chambers with fibronectin coating (ibidi, 80823) at the recommended seeding density and left overnight in a 37 °C, 5% CO_2_ incubator. The following day, cells were infected with CtrL2 with an MOI of 5. At 2 h post-infection, the cells were transfected with either vinculin-venus (62), a gift from Martin Schwartz (Addgene, 27300); paxillin-pEGFP (63), a gift from Rick Horwitz (Addgene, 15233); or FAK-GFP (15, 62), a gift from Kenneth Yamada (Addgene, 50515), using Lipofectamine 3000 transfection reagent (Thermo Fisher Scientific, L3000008), following the manufacturer's instructions. After 20–22 h, time-lapsed images of transfected cells were obtained using a Leica SD6000 AF in TIRF mode at the Washington State University IPN Imaging Core. Images of the GFP-tagged proteins were collected every minute for 90 min. The time-lapse images were uploaded to the Focal Adhesion Analysis Server (63).

### Cell motility assay

MEFs were seeded on ibidi μ-slide live-cell imaging chambers (ibidi, 80426). Cells were infected with *C. trachomatis* serovar L2 at an MOI of 10 by rocking at 4 °C for 1 h. Infected cells were imaged starting at 20 h post-infection. A mock-infected control was made following the same protocol but without *Chlamydia* infectious particles. Cells were transfected with N1-mTurquoise2 empty vector control or TarP^829–1006^-mTurquoise2 (LDVBD) using electroporation, seeded into an ibidi μ-slide, and imaged starting at 22 h post-electroporation. Cells were imaged in live-cell imaging solution (Thermo Fisher Scientific, A14291DJ) supplemented with 5% fetal bovine serum within a 37 °C, 5% CO_2_ controlled environment. Time-lapse DIC images were obtained using a Leica SD6000 AF microscope every 10 min for 10 h. To minimize the risk of phototoxicity, we restricted image acquisition of the fluorescent mTurquoise2 channel for our transfected cells to the last frame alone. We utilized a similar individual cell-tracking data analysis approach as described previously (64). Cell motility was tracked using the manual tracking function in ImageJ. Each individual cell was tracked using the position of the nucleus over time. We maximized the time of analysis for each experiment based on the number of cells that remained within a trackable field of view over the imaging span. The coordinate data from the manual tracking function were uploaded to ibidi's chemotaxis and migration tool. Measurements were taken from spatially calibrated images with a (pixel/μm) scale contained within the metadata. The *x*/*y* calibration was set to 0.800001 based on the microscope's settings contained within the file's metadata. The statistics function was used to determine the velocity and Euclidean (straight-line) distance traveled for each cell.

### De novo protein inhibition

COS7 cells were cultured as described previously. Prior to infection, cells and EB particles were treated with 60 μg/ml chloramphenicol (Sigma, C0378) for 30 min. Cells and EBs were kept in chloramphenicol-supplemented DMEM throughout infection until fixation. Cells were fixed at 8 or 20 h post-infection and were immunostained as described above. For quantification, images were submitted to the Focal Adhesion Analysis Server, which provided an area value for each adhesion within the image. For each treatment, >850 individual FAs were analyzed. The minimum adhesion size was set at 10 pixels when submitting to the server, which was the minimum value found to differentiate adhesions from cytosolic background.

### Western blotting

COS7 cells were plated in 6-well plates with duplicate wells and incubated at 37 °C and 5% CO_2_ until 80% confluence. Cells were infected, as described previously, with CtrL2 for 0 min, 8 h, or 24 h with an MOI of 200. Proteins were harvested using ice-cold radioimmune precipitation assay buffer (Millipore, 20-188) supplemented with phosphatase (Sigma, 4906845001) and protease inhibitors (Sigma, 5892970001). Cells were scraped and incubated for 30 min on ice. The lysates were centrifuged at 13,000 × *g* for 20 min at 4 °C. Supernatants were diluted in Laemmli buffer (Bio-Rad, 161-0747) and kept at −20 °C before analysis. Samples were resolved on 10% acrylamide SDS-PAGE. Proteins were transferred to a nitrocellulose membrane (Bio-Rad, 1620115). Immunoblotting was performed by blocking membranes with 5% BSA in TBS-T overnight at 4 °C and incubation using antibodies against TarP (a generous gift from Dr. Raphael Valdivia, Duke University) and β-tubulin horseradish peroxidase–conjugated (Abcam, ab21058). The secondary antibody used was anti-mouse horseradish peroxidase–conjugated (DAKO P0161). Immobilin chemiluminescence kit (Millipore, WBKLS0500) was used to develop the blot.

### Cloning and transfection of TarP constructs

A summary of the primers used in this study is provided in Table S1. Initially, TarP full-length, TarP LDVBD, TarP LD, and TarP VBD were PCR-amplified from CtrL2 genomic DNA using the primer combinations 1-2, 5-2, 5-6, and 4-2, respectively. BamHI (reverse primer) and KpnI (forward primer) restriction sites were used for fusion with the N1-mTurquoise2 plasmid. The TarP ΔPRD was obtained using the 7-8 primer pair for PCR amplification from TarP full-length-mTurquoise2 fusion plasmid. The primers were created to amplify the whole TarP full-length-mTurquoise2 except the nucleotides that constitute the PRD, 625–650. The resulting PCR product was recombined using in-Fusion HD cloning plus CE (Clontech, 638916) to create a functional circular plasmid. TarP ΔLDVBD was PCR-amplified from the TarP ΔPRD-mTurquoise2 plasmid using the primer pair 1-3. The same restriction enzymes were used to clone these fragments into N1-mTurquoise2. Transformations using restriction enzyme recombination were made into chemically competent Top10 (Invitrogen) *E. coli*, and vector sequences was verified using sequencing (Eurofins). The constructs pFH-TarP ΔPRD and pFH-TarP ΔLDVBD used for superresolution experiments were PCR-amplified. The vector backbone 1436 pcDNA3-FLAG-HA, kindly provided by William Sellers (Addgene, 10792), was linearized by PCR using the primer pairs 11-12 and 11-18, creating homology overhang regions to the TarP ΔPRD and TarP ΔLDVBD, respectively. TarP LDVBD was amplified from CtrL2 genomic DNA using the primer pair 13-14 and was cloned into 1436 pcDNA3-FLAG-HA linearized by PCR using the primer pair 15-16. Fragments and vector backbone were recombined using in-Fusion HD cloning plus CE (Clontech, 638916) to create a functional circular plasmid and transformed into chemically competent Stellar *E. coli* (Clontech). Vectors were verified by Sanger sequencing (Eurofins). The pcDNA3-FLAG-Apex-Nes was a gift from Alice Ting (Addgene, 49386). The N1-mTurquoise2 was a gift from Michael Davidson and Dorus Gadella (Addgene, 54843). For iPALM experiments 1 μg of DNA and 2 μl of sheared salmon sperm DNA were mixed together in 15 μl of Opti-MEM (Thermo Fisher Scientific, 31985062) and kept on ice for 15 min. 1 × 10^6^ COS7 cells were resuspended in 200 μl of cold Opti-MEM, mixed with the DNA solution, and kept on ice for 30 s. Cells and DNA suspension were transferred to a 4-mm gap cuvette (Bio-Rad, 1652088) and electroporated using Bio-Rad Gene Pulser XCell using the following settings: 190 V, 950 microfarads, infinity. After electroporation, 1.5 ml of warm growth medium was added. 400 μl of cell solution was added to a 6-well plate well containing 1.5 ml of warm growth medium and the gold fiducial coverslip. Cells were incubated at 37 °C and 4% CO_2_ for 4 h to adhere to the gold fiducial coverslip. Cells were washed to remove dead cells debris and further incubated for 20 h.

### iPALM imaging and analysis

iPALM imaging and analysis were performed as described previously (38, 65) with the following modifications. After 24 h of transfection cells plated in gold fiducial coverslip were fixed with 0.8% PFA and 0.1% glutaraldehyde (Sigma, G7526-10ML) solution (in PBS) for 10 min. After fixation, cells were washed three times with PBS and quenched using 1% NaBH_4_ (Sigma, 452882-25G) solution (in PBS) for 7 min. Cells were then washed again three times with PBS. After washing, cells were immunostained (when necessary) and/or processed for iPALM imaging as described previously (38). The vertical coordinates relative to the gold fiducial markers are indicated by a color scale from red (0 nm) to purple (250 nm).

### Trypsin assay

Cells were plated in 24-well plates and incubated at 37 °C and 5% CO_2_ until 80–90% confluence. Afterward, cells were infected with CtrL2 with a multiplicity of infection of 5 for 20 h. Cells were then treated with 0.01% trypsin diluted in serum-free DMEM at 37 °C, for 0, 10, 20, 30, or 35 min. Cells were fixed with 4% PFA, carefully washed with PBS, and stained with DAPI to count the number of remaining cells as well as to visualize *Chlamydia* inclusions. Images were taken using a Nikon Eclipse TE2000-U microscope. Detachment assays were performed as above, and transfection procedures were as described.

### Zyxin assay

RFP-zyxin was a gift from Anna Huttenlocher (Addgene plasmid 26720) (66). For the blebbistatin assay, cells were transfected with RFP-zyxin using electroporation and allowed to adhere overnight. Cells were then mock-infected or CtrL2-infected with an MOI of 10. At 20 h post-infection, cells were treated with 10 μm blebbistatin. Cells were fixed with 4% paraformaldehyde at 5-, 15-, and 30-min time points post-treatment and prepared for immunofluorescence as described previously. Paxillin was visualized using a rabbit mAb (Abcam, ab32084). For the quantitative comparison of paxillin- and zyxin-positive adhesions, cells were either transfected with RFP-zyxin or co-transfected with RFP-zyxin and the empty vector or LDVBD-mTurquoise2 and immunostained for paxillin. To count the number of focal adhesions in the zyxin and paxillin channel, each channel was separately uploaded to the Focal Adhesion Analysis Server to create a mask. The adhesions were then counted using the particle-counting function in ImageJ.

## Data availability

Data are contained in the article as well as the supporting information. iPALM data are available upon request.

## Supplementary Material

Supporting Information
