# Supplementary material for: A post-invasion role for Chlamydia type III effector TarP in modulating the dynamics and organization of host cell focal adhesions
Source: J Biol Chem. 2020 Aug 25;295(43):14763–79. doi: 10.1074/jbc.RA120.015219 (PMC7586217; doi:10.1074/jbc.RA120.015219)
Supplement: Supporting Information [file supp_295_43_14763__index.html]

A post-invasion role for Chlamydia type III effector TarP in modulating the dynamics and organization of host cell focal adhesions — Chlamydia modulates focal adhesion stability — A post-invasion role for Chlamydia type III effector TarP in modulating the dynamics and organization of host cell focal adhesions — Chlamydia modulates focal adhesion stability — Supporting Information 

# A post-invasion role for *Chlamydia* type III effector TarP in modulating the dynamics and organization of host cell focal adhesions

## Supporting Information

- Movie S1 - Motility of mock-infected cells
- Movie S2 - Motility of Chlamydia-infected cells
- Movie S3 - Motility of cells transfected with vector only
- Movie S4 - Motility of cells expressing the TarP LDVBD domains
- Movie S5 - Detachment of mock-infected cells during trypsinization
- Movie S6 - Detachment of Chlamydia-infected cells during trypsinization
- Supplemental Figures - Supplemental Table 1 and Figures S1-S5
