# Supplementary material for: A post-invasion role for Chlamydia type III effector TarP in modulating the dynamics and organization of host cell focal adhesions
Source: J Biol Chem. 2020 Aug 25;295(43):14763–79. doi: 10.1074/jbc.RA120.015219 (PMC7586217; doi:10.1074/jbc.RA120.015219)
Supplement: Supporting Information [file supp_RA120.015219_162257_1_supp_580529_q5y5fg.pdf]

SUPPORTING INFORMATION

Supplemental Table 1 - Primers used in this study

Primer number	Primer name	Primer sequence 5' to 3'
1	Fwd-TarP	GCATGGTACCATGACGAATTCTATATCAGGTG
2	Rev-VBD domain	TAATGGATCCGGGTATCCTACGGTATCAATC
3	Rev- TarP$^{1\text{-}829\Delta625\text{-}650}$	TAATGGATCCCTTGGAGGTGGTGAAGG
4	Fwd-VBD domain	TAATGGTACCATGAACAAATTCCGCAAAGAA
5	Fwd-LD domain	TAATGGTACCATGACCCCATCAACTACAACA
6	Rev-LD domain	TAATGGATCCATAGATCCCGGCTTGCC
7	FWD-TarPΔ$^{625\text{-}650}$	ATGGCATTGTCAATGTCAACGTTGGC
8	Rev-TarPΔ$^{625\text{-}650}$	ACATTGAAATGCCATCGTCTTCGCT
9	Fwd-TarP-FH	AACGGCCGCCAGTGTATGACGAATTCTATATCAGGTG ATCAACCT
10	Rev-TarP-FH	CCCTCTAGATGCATGTTATCCTACGGTATCAATCAGT GAGCTT
11	Fwd-FH-TarP	GATACCGTAGGATAACATGCATCTAGAGGGCCCTATT CTATAG
12	Rev-FH-TarP	TATAGAATTCGTCATACACTGGCGGCCGTTACTA
13	Fwd-TarP$^{829\text{-}1006}$-FH	AACGGCCGCCAGTGTACCCCATCAACTACAACATTAA GAACG
14	Rev- TarP$^{829\text{-}1006}$-FH	CCCTCTAGATGCATGTCCTACGGTATCAATCAGTGAG CTTAG
15	Fwd-FH- TarP$^{829\text{-}1006}$	ATTGATACCGTAGGACATGCATCTAGAGGGCCCTATT CTATAG
16	Rev-FH- TarP$^{829\text{-}1006}$	TGTAGTTGATGGGGTACACTGGCGGCCGTTACTA
17	Rev-TarP$^{1\text{-}829\Delta625\text{-}650}$-FH	CCCTCTAGATGCATGTTATGGAGGTGGTGAAGGCAG TAG
18	Fwd-FH- TarP$^{1\text{-}829\Delta625\text{-}650}$	CCTTCACCACCTCCATAACATGCATCTAGAGGGCCCT ATTCTATAG

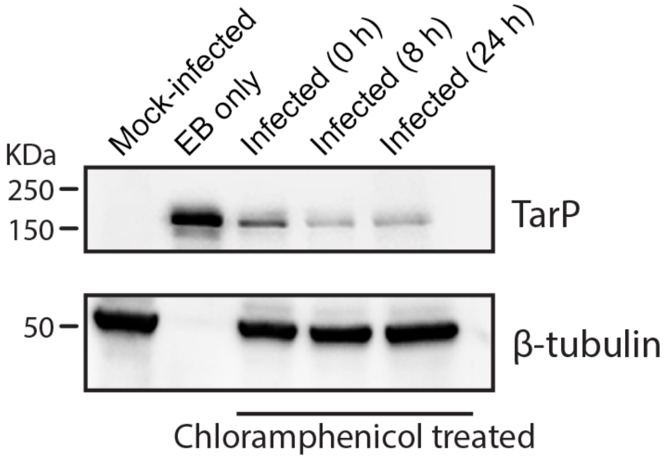

Figure S1. TarP translocated by *C. trachomatis* is stable. (A) Cos7 cells were either mock-infected or *Chlamydia*-infected for the indicated times, and maintained throughout in the presence of the prokaryotic protein synthesis inhibitor chloramphenicol. Whole cell lysates were harvested to monitor by Western blot the presence and stability of translocated TarP proteins. A rabbit polyclonal anti-TarP antibody was used. Protein concentrations were determined and adjusted to ensure equal loading. β-tubulin was used as the loading control. Mock-infected and EB-only samples were added to demonstrate specificity of the anti-TarP antibody used.

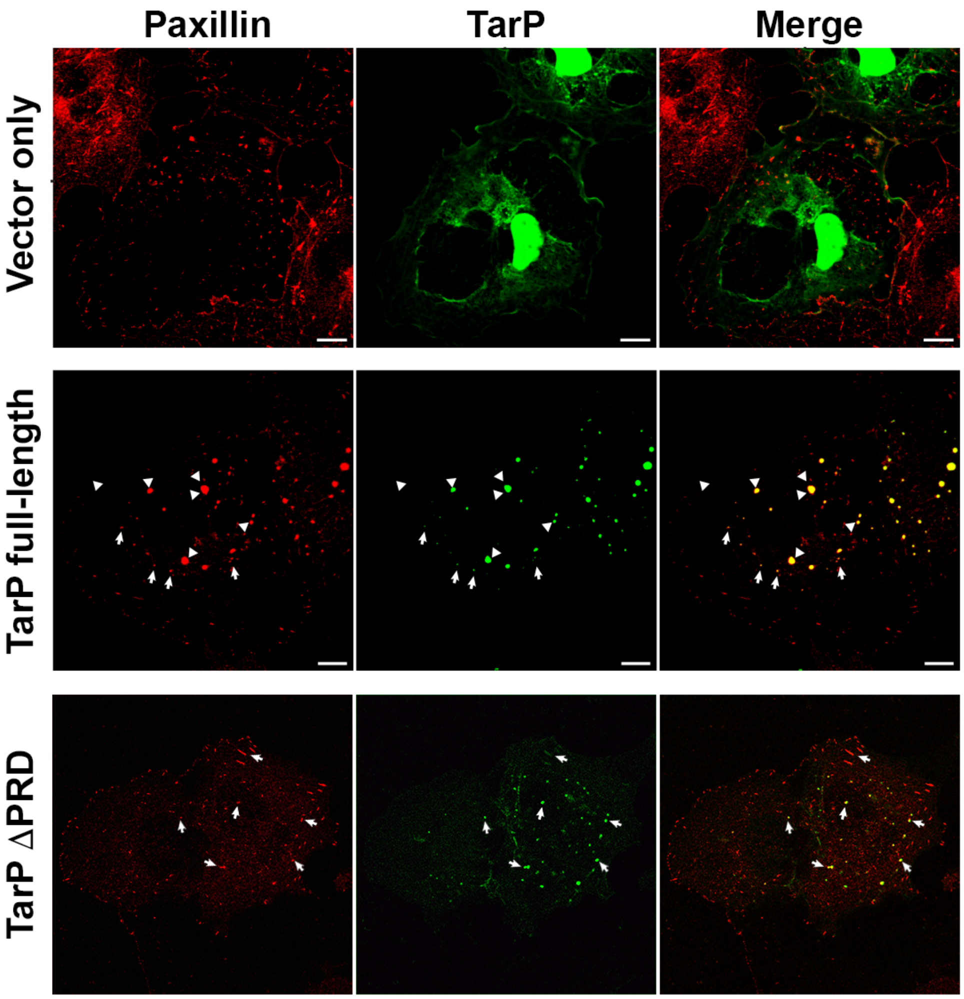

Figure S2. The PRD domain of TarP is not required for focal adhesion localization upon ectopic expression. Full-length TarP and TarP ΔPRD, both fused to mTurquoise2 were expressed ectopically in wild type MEFs for 19 h. The cells were processed for immunostaining for paxillin to visualize focal adhesions. Both full-length TarP and TarP ΔPRD colocalized with paxillin-positive structures, forming larger protein aggregates (arrowheads). Smaller TarP-positive punctae are indicated by white arrows.

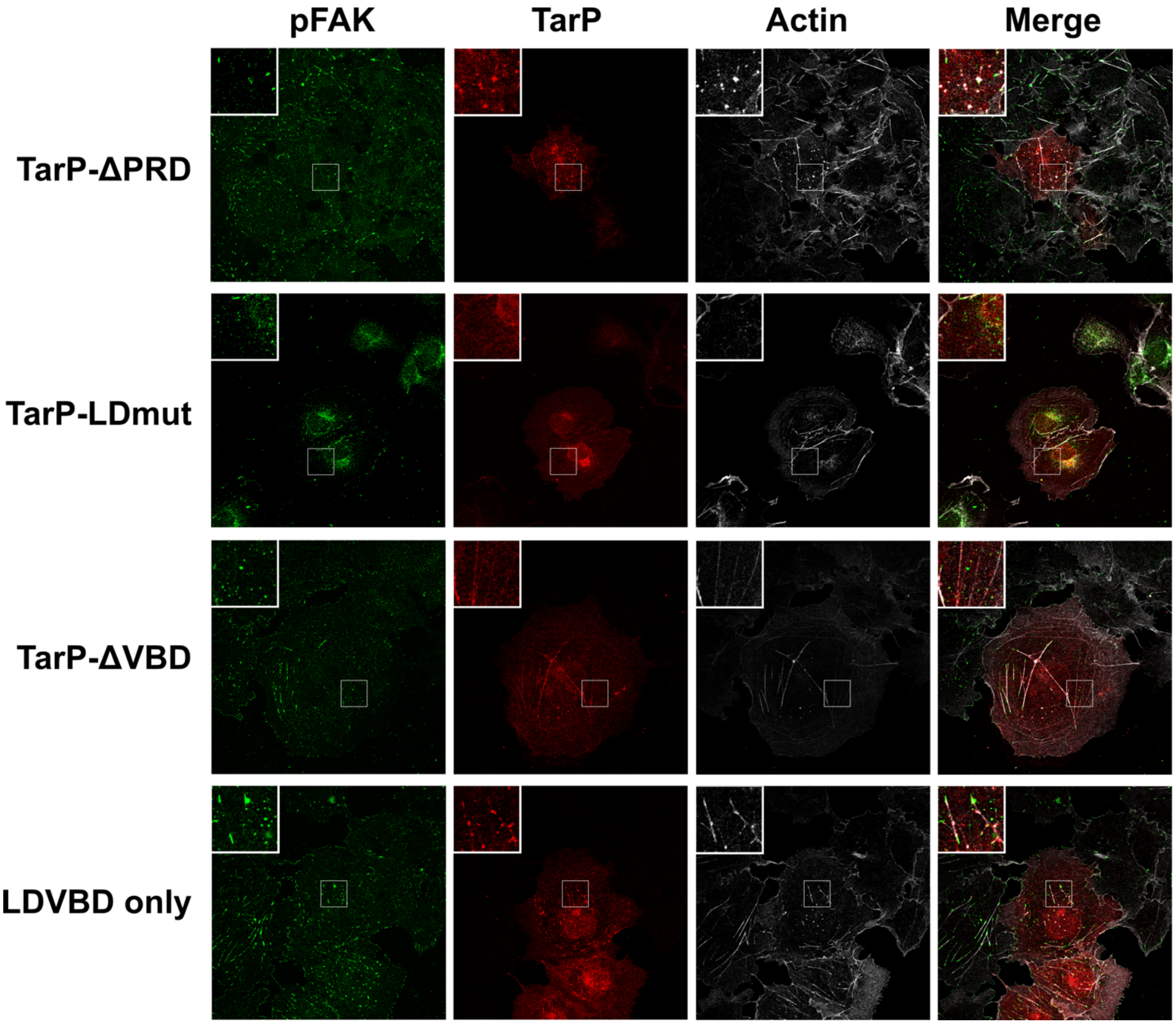

Figure S3. The LD and VBD domains are both required for FA localization of TarP. Cos7 cells were transfected to express TarP ΔPRD (wt), TarP LDmut, TarP ΔVBD, or LDVBD only. All proteins were expressed as HA-tagged fusions. At 18 h post-transfection, the cells were processed for immunofluorescence confocal imaging. Focal adhesions, TarP constructs, and actin stress fibers, were visualized using monoclonal antibodies to pFAK and hemagglutinin (HA)-tag and phalloidin, respectively. Note the redistribution of TarP LDmut and TarP ΔVBD from FAs to stress fibers. Regions of interest are bounded by white lines.

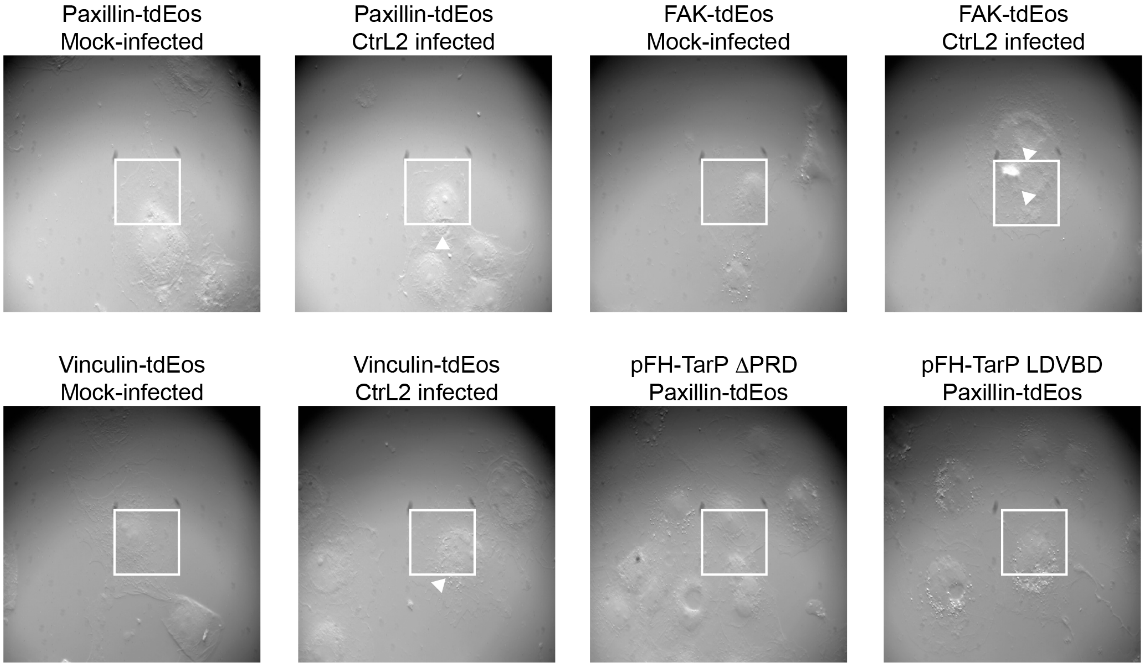

Figure S4. Infection disrupts the stratified organization of focal adhesions. Cos7 cells were either mock-infected or infected with *C. trachomatis* serovar L2 for 20 h. DIC images of cells analyzed by iPALM were acquired to demonstrate the infection state. Regions of interests are bounded by white lines, and inclusions indicated by arrowheads.

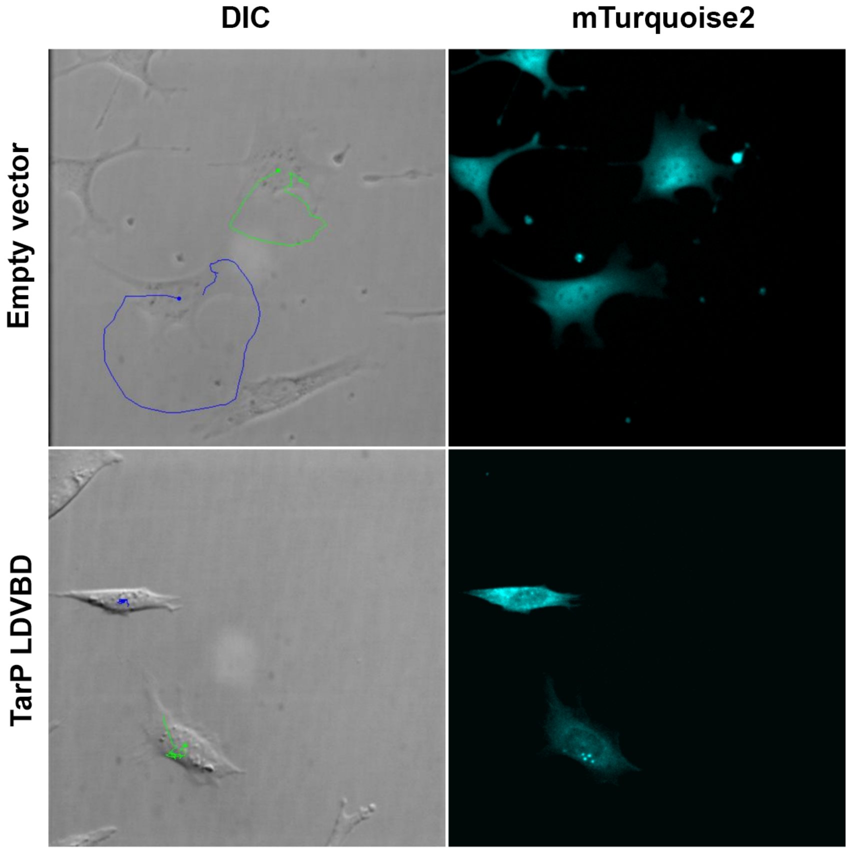

Figure S5. LDVBD-expressing cells exhibited restricted motility relative to the empty vector-transfected control cells. Acquisition of the fluorescent channel was limited to the final frame of the motility assay to minimize phototoxicity. Images show the final DIC and fluorescent channel captured following 10 hours of time-lapse imaging. The DIC channel includes the dot and line overlay generated via ImageJ's manual tracking function to indicate the cell's movement over time.

Movie S1. Infection inhibits cell motility. A representative video assembled from a 5-h time-lapse imaging of mock-infected MEFs shows motility of individual cells with track outlines included.

Movie S2. Infection inhibits cell motility. A representative video assembled from a 5-h time-lapse imaging of *C. trachomatis*-infected MEFs shows motility of individual cells with track outlines included.

Movie S3. LDVBD is sufficient to inhibit cell motility. MEFs transfected with the vector alone were monitored for 10 h. A representative video assembled from a series of time-lapse images shows the degree of cell motility in the vector-only control group.

Movie S4. LDVBD is sufficient to inhibit cell motility. MEFs transfected with the LDVBD-mTurquoise2 were monitored for 10 h. A representative video assembled from a series of time-lapse images shows inhibition of cell motility of LDVBD-expressing cells.

Movie S5. *Chlamydia*-infected cells are resistant to detachment by mild trypsinization. Mock-infected HeLa cells growing on glass coverslips were treated with 0.025% Trypsin + EDTA, and imaged at 60-s intervals for 30 min. Note that the cells start to round up by seven min of incubation in trypsin.

Movie S6. *Chlamydia*-infected cells are resistant to detachment by mild trypsinization. *C. trachomatis* serovar L2-infected HeLa cells growing on glass coverslips were treated with 0.025% Trypsin + EDTA at 24 h post-infection, and imaged at 60-s intervals for 30 min. In contrast to mock-infected cells shown in Movie S5, the infected cells remained attached and spread out after 30 min of mild trypsinization, indicating a possible enhancement of adhesion of infected cells to the substrate.
